# Supplementary material for: Enhancing Antimicrobial Peptides from Frog Skin: A Rational Approach
Source: Biomolecules. 2025 Mar 20;15(3):449. doi: 10.3390/biom15030449 (PMC11939955; doi:10.3390/biom15030449)
Supplement: Supplementary file 1 [file biomolecules-15-00449-s001.zip › biomolecules-3511673-supplementary.pdf]

SUPPORTING INFORMATION

RESULTS

RP-HPLC chromatograms of synthetic peptides.

All synthetic peptides were purified. The monoisotopic molecular masses were determined as *m/z* 925.5935, 1734.437, 2058.70, 2089.487, 2157.597, 2172.7 and 2215.800 for hylin-Pul3, dHP3-31, dHP3-50, dHP3-50.137, dHP3-50.190, dHP3-84 and dHP3-84.39 peptides, respectively.

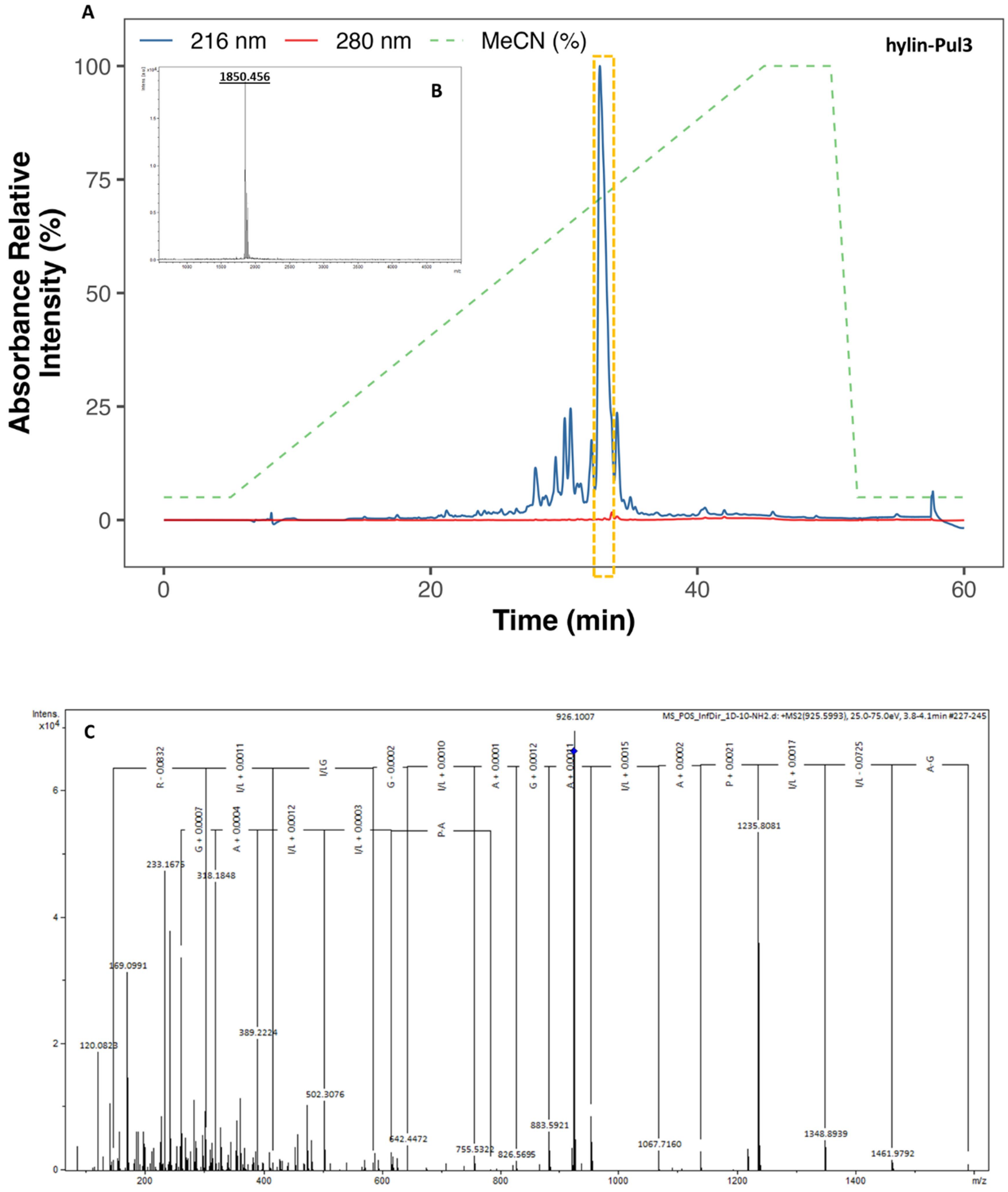

Figure S1. (**A**) HPLC chromatogram of synthetic peptide hylin-Pul3 monitored at 216 and 280 nm. Selected peak was collected for purification and lyophilization. (B) MALDI-TOF

MS analysis of the main chromatographic fraction after purification procedures. The experimental monoisotopic molecular mass was determined by high resolution mass spectrometry as 1850.1797 Da, corroborating the peptide content (theoretical mass = 1850.1738 Da). (C) Fragmentation spectrum from the double charged ion at *m/z* 925.5935 obtained by electrospray ionization-quadrupole-time-of-flight (ESI-QTOF) MS showing annotated amino acids after *de novo* sequencing. K/Q and L/I ambiguities are not resolved here.

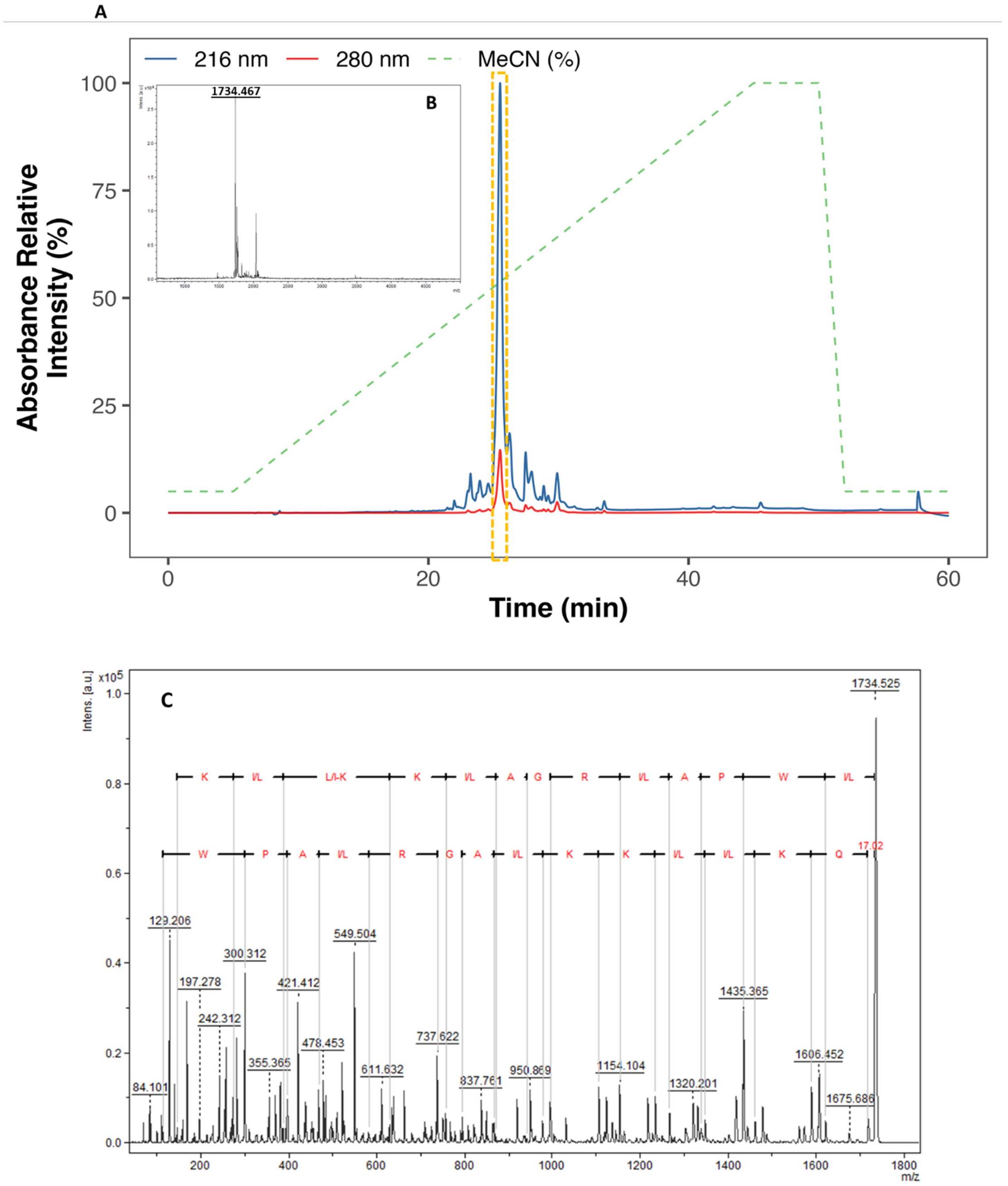

Figure S2. (**A**) HPLC chromatogram of synthetic peptide dHP3-31 monitored at 216 and 280 nm. Selected peak was collected for purification and lyophilization. (B) MALDI-TOF MS analysis of the main chromatographic fraction after purification procedures. The experimental monoisotopic molecular mass was determined by high resolution mass spectrometry as 1734.1629 Da, corroborating the peptide content (theoretical mass = 1734.1653 Da). (**C**) Fragmentation spectrum from the ion at *m/z* 1734.44 obtained by MALDI-TOF/TOF MS/MS

showing annotated amino acids after *de novo* sequencing. K/Q and L/I ambiguities are not resolved here.

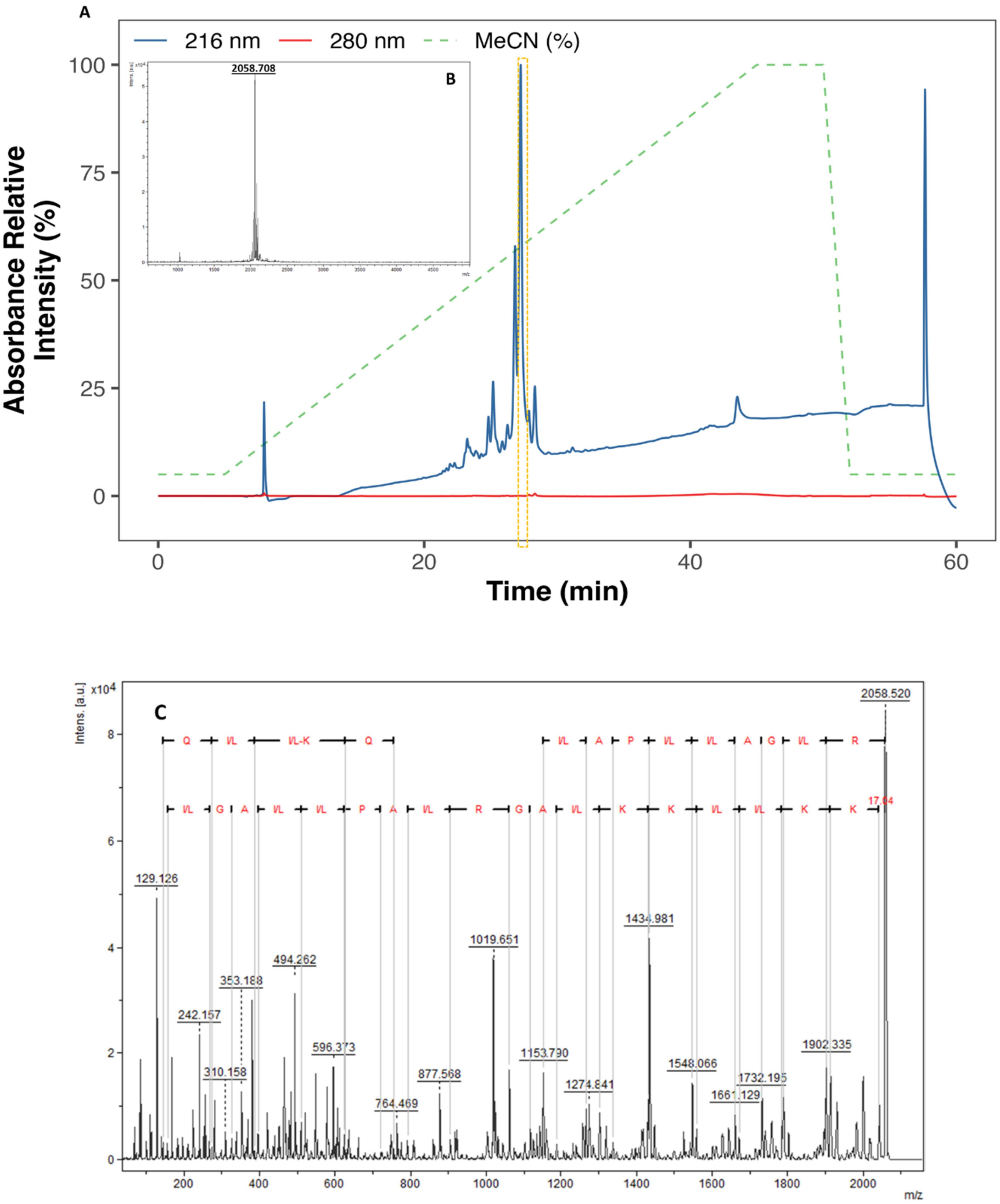

Figure S3. (**A**) HPLC chromatogram of synthetic peptide dHP3-50 monitored at 216 and 280nm. Selected peak was collected for purification and lyophilization. (B) MALDI-TOF MS analysis of the main chromatographic fraction after purification procedures. The experimental monoisotopic molecular mass was obtained by high resolution mass spectrometry as 2058.4286 Da, corroborating the peptide content (theoretical mass = 2058.4114 Da). (**C**) Fragmentation spectrum from the ion at *m/z* 2058.71 obtained by MALDI-TOF/TOF MS/MS showing annotated amino acids after *de novo* sequencing. K/Q and L/I ambiguities are not resolved here.

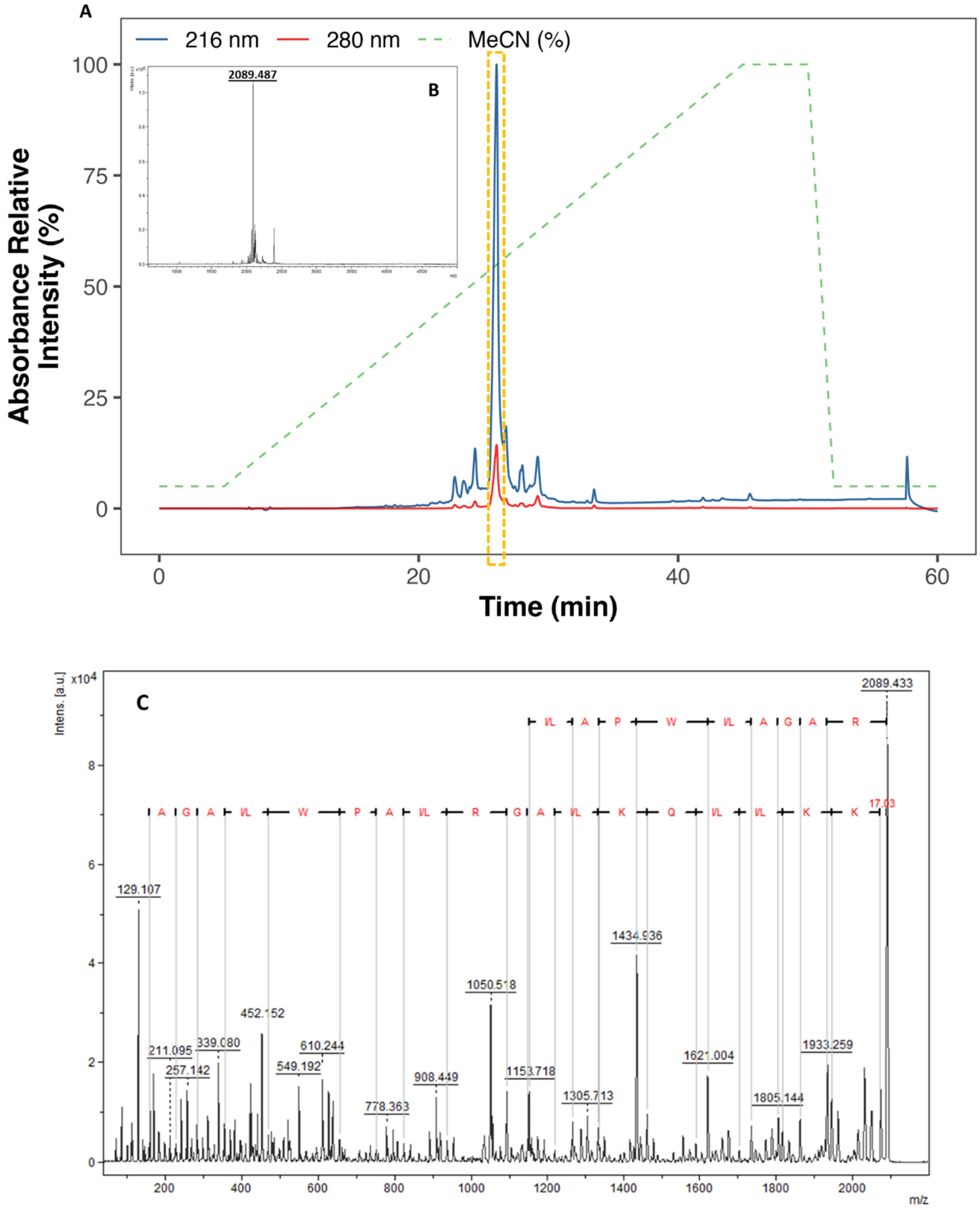

Figure S4. (**A**) HPLC chromatogram of synthetic peptide dHP3-50.137 monitored at 216 and 280nm. Selected peak was collected for purification and lyophilization. (B) MALDI-TOF MS analysis of the main chromatographic fraction after purification procedures. The experimental monoisotopic molecular mass was determined by high resolution mass spectrometry as 2089.3743 Da, corroborating the peptide content (theoretical mass = 2089.3597 Da). (C) Fragmentation spectrum from the ion at m/z 2089.49 obtained by MALDI-TOF/TOF MS/MS showing annotated amino acids after de novo sequencing. K/Q and L/I ambiguities are not resolved here.

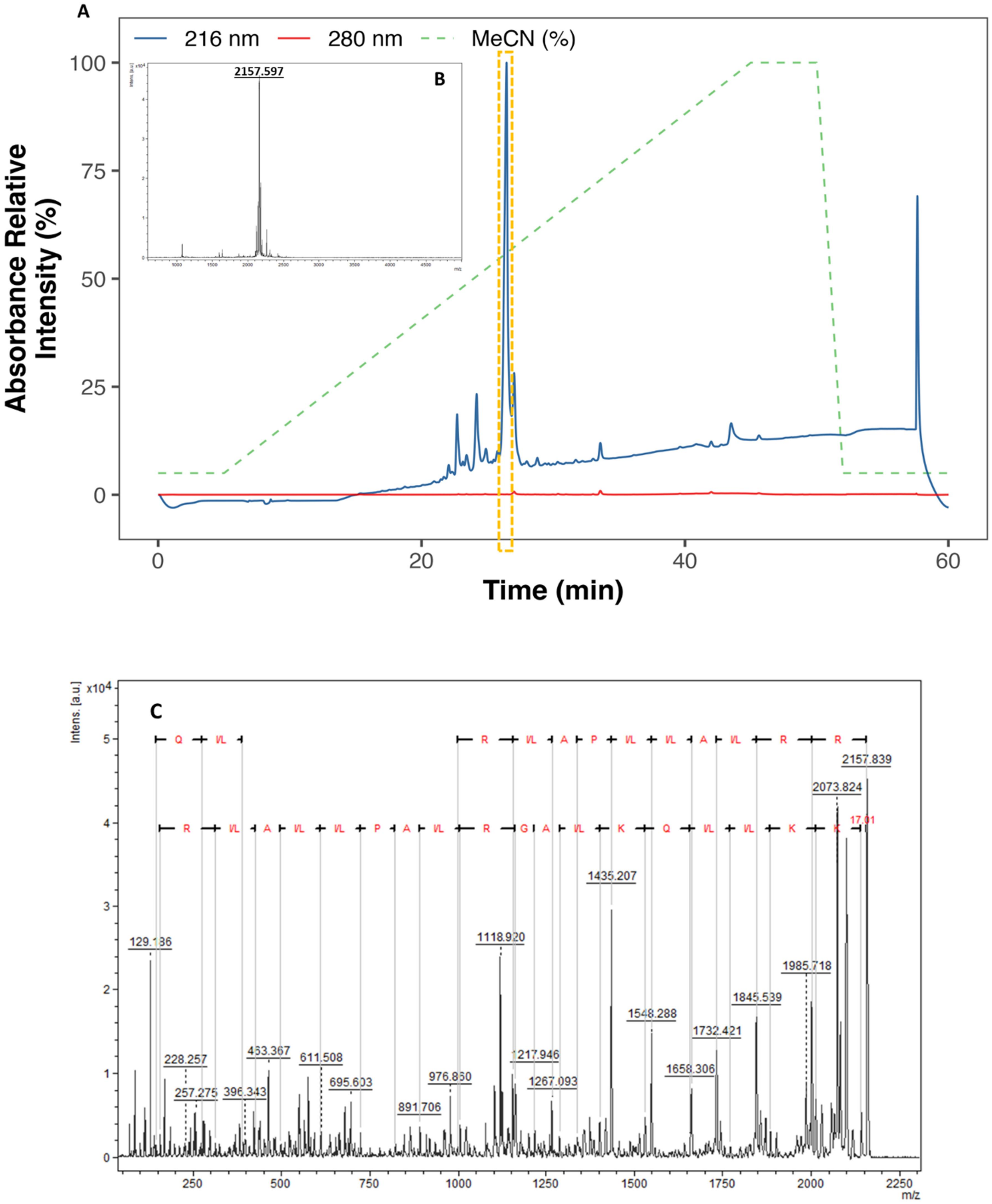

Figure S5. (**A**) HPLC chromatogram of synthetic peptide dHP3-50.190 monitored at 216 and 280nm. Selected peak was collected for purification and lyophilization. (B) MALDI-TOF MS analysis of the main chromatographic fraction after purification procedures. The experimental monoisotopic molecular mass was determined by high resolution mass spectrometry as 2157.5019 Da, corroborating the peptide content (theoretical mass = 2157.4910 Da). (C) Fragmentation spectrum from the ion at *m/z* 2157.597 obtained by MALDI-TOF/TOF MS/MS showing annotated amino acids after *de novo* sequencing. K/Q and L/I ambiguities are not resolved here.

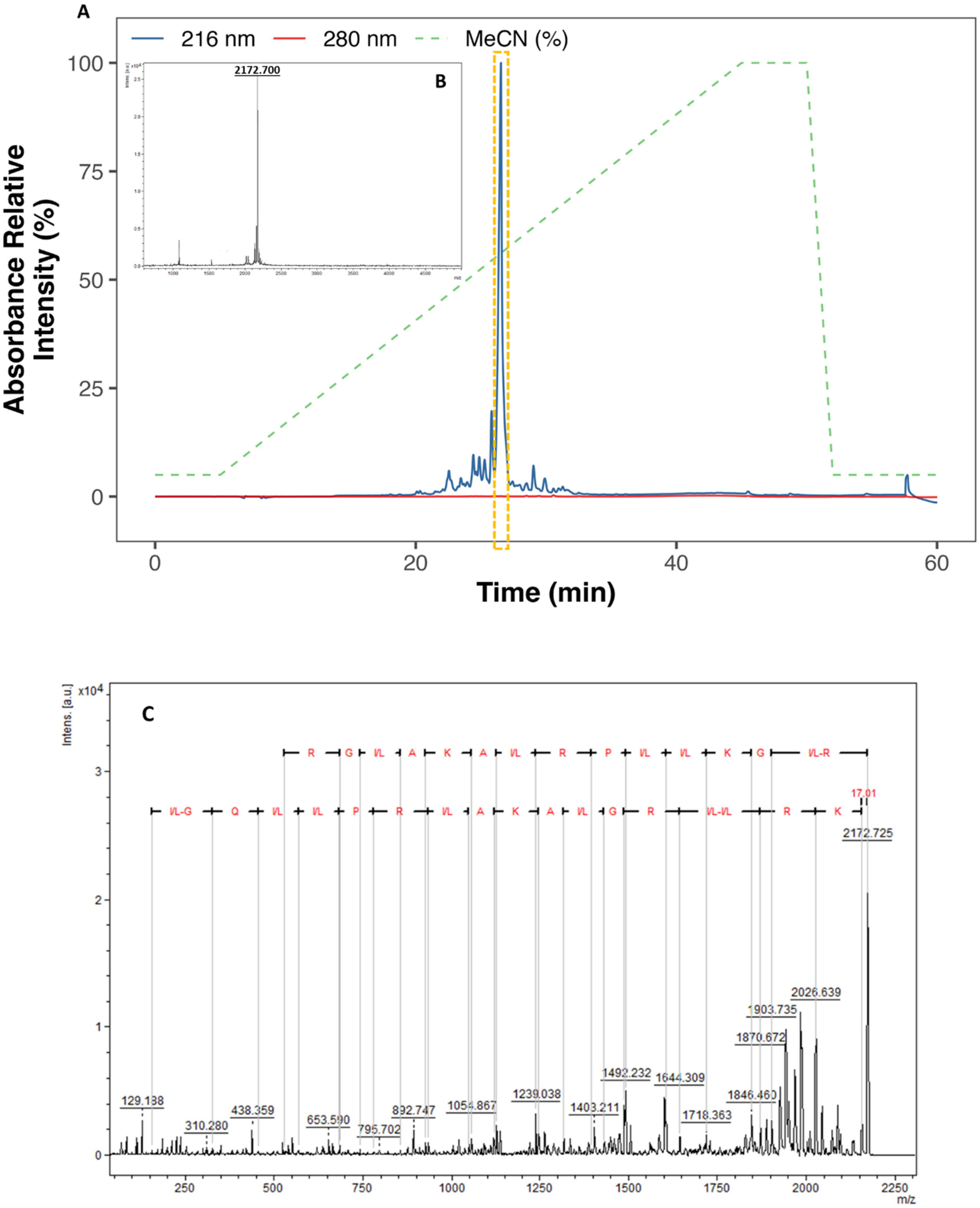

Figure S6. (**A**) HPLC chromatogram of synthetic peptide dHP3-84 monitored at 216 and 280nm. Selected peak was collected for purification and lyophilization. (B) MALDI-TOF MS analysis of the main chromatographic fraction after purification procedures. The experimental monoisotopic molecular mass was determined by high resolution mass spectrometry as 2172.476 Da, corroborating the peptide content (theoretical mass = 2172.4655 Da). (C) Fragmentation spectrum from the ion at *m/z* 2172.70 obtained by MALDI-TOF/TOF MS/MS showing annotated amino acids after de novo sequencing. K/Q and L/I ambiguities are not resolved here.

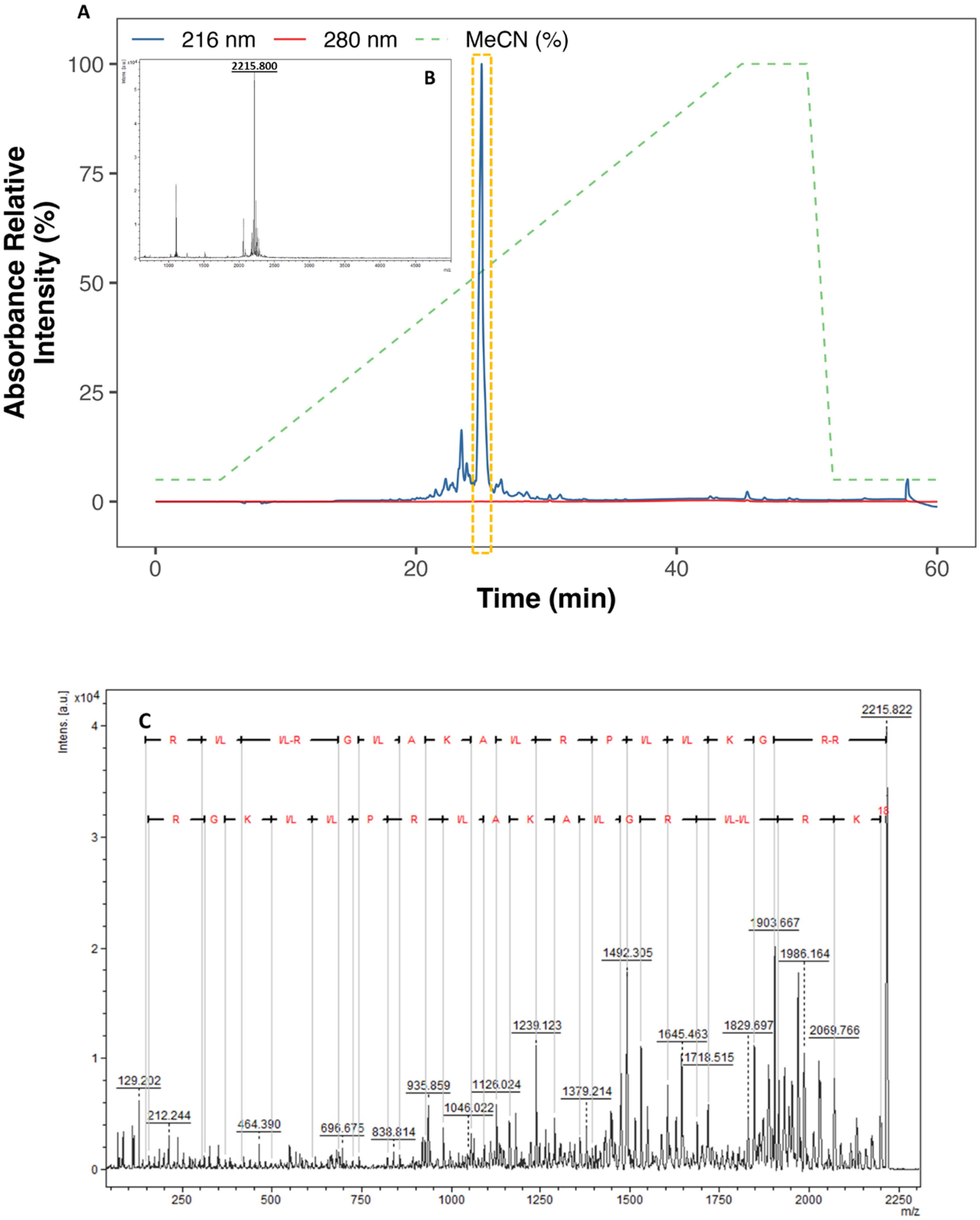

Figure S7. (A) HPLC chromatogram of synthetic peptide dHP3-84.39 monitored at 216 and 280nm. Selected peak was collected for purification and lyophilization. (B) MALDI-TOF MS analysis of the main chromatographic fraction after purification procedures. The experimental monoisotopic molecular mass was determined by high resolution mass spectrometry as 2215.4871 Da, corroborating the peptide content (theoretical mass = 2215.4826 Da). (C) Fragmentation spectrum from the ion at *m/z* 2215.80 obtained by MALDI-TOF/TOF MS/MS showing annotated amino acids after de novo sequencing. K/Q and L/I ambiguities are not resolved here.

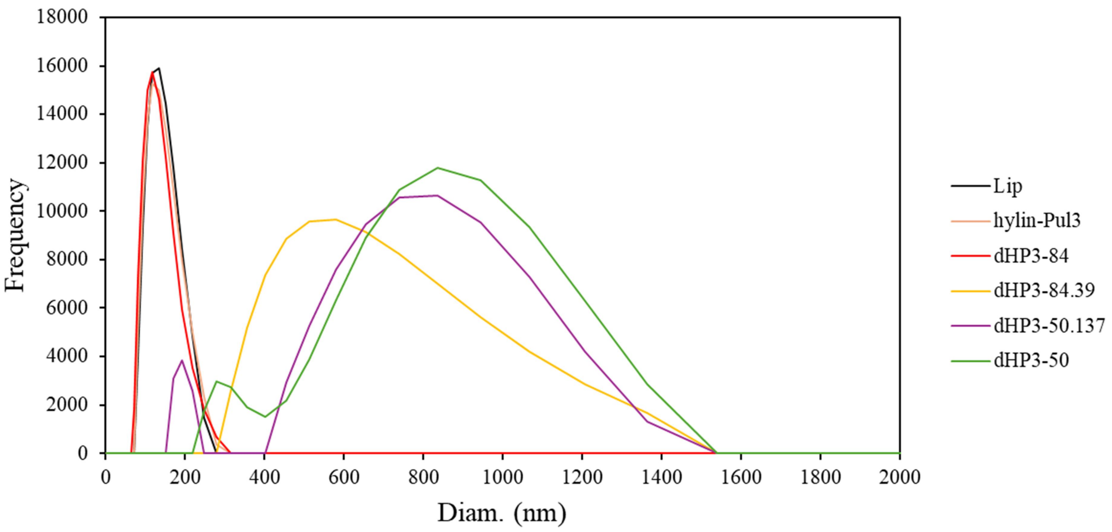

Figure S8: Dynamic light scattering of peptides hylin-Pul3, dHP3-84, dHP3-50, dHP3-50.137, and dHP3-89.39 interacting with liposomes composed of *E. coli* total lipid extract. Size distribution of liposomes membranes (black) and liposomes after the addition of 50 µM of each peptide (colored traces).

Table S1. *In silico* characterization of selected peptides, including predictions of their antimicrobial and hemolytic activities. A: active; NA: not active; H: hemolytic; NH: Non-hemolytic.

		hylin-Pul3	dHP3-31	dHP3-50	dHP3-50.137	dHP3-50.190	dHP3-84	dHP3-84.39
In silico characterization	**Length**	19	15	19	19	19	19	19
	**Theorical Molecular Weight** (g/mol)	1850.3	1734.2	2058.6	2018.2	2157.7	2172.7	2215.7
	**Chargue**	3	6	7	7	8	7	8
	**Hydrophobic ratio** (%)	68	60	57	57	57	52	47
	**Helicity percentage** (%)	79	80	73	68	63	78	68
	**Hydrophobicity** (<H>)	0.74	0.50	0.42	0.38	0.37	0.35	0.20
	**Hydrophobic Moment** (<µH>)	0.50	0.65	0.58	0.3	0.43	0.75	0.61
In silico prediction	*ANTIMICROBIAL PREDICTION*							
	**CAMPr3** (Prob)	0.92	0.99	0.97	0.89	0.98	0.95	0.89
	**DBAASP -** *E. coli*	A	A	A	A	A	A	A
	**DBAASP-** *S. aureus*	NA	A	A	A	A	A	A
	HEMOLYTIC PREDICTION							
	**HAPPEN** (Prob)	0.91	0.44	0.47	0.46	0.29	0.30	0.05
	**HemoPred**	H	NH	NH	NH	NH	NH	NH

**DBAASP** (Erytrocites)	A	NA	NA	NA	NA	NA	NA

Table S2. Determination of Antioxidant, cytotoxic and antiviral activities. MNCC: maximal non-cytotoxic concentration; CC_{50}: concentrations at which each peptide induces 50% cell death. n/a: not active; - : not evaluated.

Name	Antioxidant activity		Biological Activity			
	DPPH	ABTS	Cytotoxicity in Vero cells (μM)		Antiviral Activity	
	mg Trolox/mg	mg Trolox/mg	MNCC	CC_{50}	μM	(%)
Hylin-PUL3	n/a	n/a	13.5	43.2	10.8	0
P31	n/a	0.14	-	-	-	-
P50	n/a	n/a	-	-	-	-
P50.137	n/a	0.08	-	-	-	-
P50.190	n/a	n/a	-	-	-	-
P84	n/a	n/a	57.6	105.9	34.5	70
P84.39	n/a	n/a	-	-	-	-
